# Intermittent neonatal hypoxia elicits the upregulation of inflammatory‐related genes in adult male rats through long‐lasting programming effects

**DOI:** 10.14814/phy2.12646

**Published:** 2015-12-10

**Authors:** Ashley L. Gehrand, Mary L. Kaldunski, Eric D. Bruder, Shuang Jia, Martin J. Hessner, Hershel Raff

**Affiliations:** ^1^ Endocrine Research LaboratoryAurora St. Luke's Medical CenterAurora Research InstituteMilwaukeeWisconsin; ^2^ Max McGee National Research Center for Juvenile DiabetesDepartment of PediatricsMedical College of WisconsinMilwaukeeWisconsin; ^3^ Departments of Medicine, Surgery, and PhysiologyMedical College of WisconsinMilwaukeeWisconsin

**Keywords:** Glucose, inflammation, insulin, intermittent hypoxia, neonatal, newborn, programming

## Abstract

The long‐term effects of neonatal intermittent hypoxia (IH), an accepted model of apnea‐induced hypoxia, are unclear. We have previously shown lasting “programming” effects on the HPA axis in adult rats exposed to neonatal IH. We hypothesized that neonatal rat exposure to IH will subsequently result in a heightened inflammatory state in the adult. Rat pups were exposed to normoxia (control) or six cycles of 5% IH or 10% IH over one hour daily from postnatal day 2–6. Plasma samples from blood obtained at 114 days of age were analyzed by assessing the capacity to induce transcription in a healthy peripheral blood mononuclear cell (PBMC) population and read using a high‐density microarray. The analysis of plasma from adult rats previously exposed to neonatal 5% IH versus 10% IH resulted in 2579 significantly regulated genes including increased expression of *Cxcl1, Cxcl2, Ccl3, Il1a*, and *Il1b*. We conclude that neonatal exposure to intermittent hypoxia elicits a long‐lasting programming effect in the adult resulting in an upregulation of inflammatory‐related genes.

## Introduction

Apnea is the most common cause of neonatal hypoxia affecting about 50% of preterm births (30–31 weeks), and is usually due to immature respiratory development (Martin and Wilson 2012). Bouts of apnea cause hypoxemia and bradycardia (Stokowski 2005). Acute hypoxia exposure in premature infants results in significant metabolic and neurological challenges early in life (Martin et al. 1986; Frankel and Stevenson 1987; Miller and Martin 1992). However, the long‐term metabolic, endocrine, and immunological effects of neonatal intermittent hypoxia (IH) exposure, an accepted model of apnea‐induced hypoxia (Martin and Wilson 2012), have not been thoroughly evaluated.

We have previously shown that in the short‐term, intermittent hypoxia elicits an acute stress response from the hypothalamic‐pituitary‐adrenal (HPA) axis in rats from age postnatal day (PD) 2 to PD12. Neonatal exposure to intermittent hypoxia significantly increases neonatal plasma ACTH and corticosterone, significantly alters glucose homeostasis, and causes hyperinsulinemia and hyperglycemia (Chintamaneni et al. 2013). We have also previously examined adults exposed to neonatal intermittent hypoxia and perinatal continuous hypoxia, and have found lasting “programming” effects on the HPA axis (Raff et al. 2007; Chintamaneni et al. 2014).

In general, the long‐term effects of hypoxic exposure in the neonate are poorly studied; the majority of the research concentrates on fetal hypoxic exposure. In humans, there is a known increased risk of insulin resistance in adulthood when the fetus is exposed to maternal hypoxia, but the mechanism is unclear (Rueda‐Clausen et al. 2011). Another study has shown that prenatal hypoxia exposure can damage the hepatic parenchymal cells associated with hepatic insulin signaling, resulting in altered hepatic development, insulin sensitivity, and glucose metabolism in postnatal life (Cao et al. 2012). Recent studies in rats have shown that perinatal IH exposure can result in oxidative stress, causing a permanent immune response subsequently resulting in features of diabetes mellitus (Pae et al. 2013).

We now assess the long‐term effects of an accepted model of apnea‐induced hypoxia (Martin and Wilson 2012) using a validated transcriptional bioassay (Kaldunski et al. 2010) to study the extracellular milieu of adult rats exposed to neonatal intermittent hypoxia. This approach was used to assess the composition of plasma pooled from adult rats exposed to neonatal intermittent hypoxia through its ability to drive a distinct transcriptional response in reporter PBMCs drawn from healthy unrelated rats. Previous programming studies have linked fetal hypoxia exposure to the upregulation of inflammatory genes and pathways in children 7–10 years of age (Bakker et al. 2000). In the rat, we hypothesize that exposure to *neonatal* intermittent hypoxia will result in an increased inflammatory state in the *adult* as a result of long‐lasting programming.

## Materials and Methods

### Animal treatments and experimental protocols

#### Sprague‐Dawley rats

Sprague‐Dawley (SD) rat pups were used to generate adult rats previously exposed to neonatal normoxia (control), 5% IH, or 10% IH. Timed pregnant SD rats (Harlan, Indianapolis, IN) were obtained and housed in a standardized environment (lights on 0600–1800) and provided a standard diet and water ad libitium at Aurora St. Luke's Medical Center. They were allowed to deliver normally and their offspring were exposed to neonatal intermittent hypoxia as described below.

#### Brown Norway rats

Brown Norway (BN) rats were used to generate the reporter PBMC population and baseline plasma (autologous controls) for the plasma induced transcriptional bioassays, as described below. BN rats were bred at the Medical College of Wisconsin (MCW), housed under specific pathogen‐free conditions with standard light/dark cycles, and were fed a regular chow diet and water ad libitum.

All Federal guidelines (http://grants1/nih/gov/grants/olaw/references/phspol.htm) for use and care of laboratory animals were followed and all protocols were approved by the respective Institutional Animal Care and Use Committees.

### Neonatal intermittent hypoxia (IH) to generate adult plasma

On postnatal days (PD) 2–6, neonatal SD rats were exposed daily to six cycles each lasting five minutes of 21% (normoxic control), 5%, or 10% intermittent hypoxia (IH) daily over one hour, as described previously (Chintamaneni et al. 2013). Otherwise, pups were returned and tended to by their birth dams and weaned at PD22. Males were allowed to mature and were sacrificed at age PD114 after an overnight fast (*N* = 16). Whole blood was collected by decapitation into tubes containing EDTA and plasma was pooled and saved for subsequent analysis.

### Peripheral blood mononuclear cell cultures

Fresh peripheral blood mononuclear cells (PBMCs) from healthy male BN rats ~180 days old (*N* = 2) were isolated by density gradient centrifugation. Transcription was induced by culturing 400 *μ*L PBMCs (500 000 cells) in cell culture media (RPMI 1640 supplemented with penicillin‐streptomycin) with 100 *μ*L of plasma for 6 h at 37°C in 5% CO_2_ as described previously (Kaldunski et al. 2010; Chen et al. 2013). The following pooled plasma samples were used: (1) autologous BN plasma (self‐baseline background control); (2) adult SD normoxic (neonatal control) plasma (*N* = 8); (3) plasma from adult rats exposed to 5% neonatal IH (*N* = 5); or (4) plasma from adult rats exposed to 10% neonatal IH (*N* = 3). RNA was extracted from PBMCs using TRIzol reagent (Invitrogen, Carlsbad, CA). Each culture was assessed with a single array (2 BN × 4 conditions = 8 chips).

### Gene chip analysis

The methodological details and validation of this analysis have been described previously (Kaldunski et al. 2010). Briefly, RNA from PBMCs was amplified/labeled using the Express IVT kit (Affymetrix, Santa Clara, CA), and hybridized to the Affymetrix Rat Genome 230 2.0 Array. Image data were quantified with Affymetrix Expression Console Software and normalized with Robust Multichip Analysis (www.bioconductor.org) to determine signal log ratios. Rank Product (RP) *P*‐values and false discovery rates (FDRs) were determined using RankProd from Bioconductor (http://www.bioconductor.org/packages/release/bioc/html/RankProd.html). Rank Product is a nonparametric statistic that detects items that are consistently highly ranked within a list. This was used to identify differentially expressed probe sets based on the estimated percentage of false predictions (Hong et al. 2006). Differentially expressed probe sets were defined as those possessing a *P*‐value <0.05 and ¦log_2_ ratio¦ > 0.263 between the compared groups. Ontological analysis was performed with the Database for Annotation, Visualization, and Integrated Discovery (DAVID) (http://david.abcc.ncifcrf.gov/) (Huang et al. 2009) and the Ingenuity Pathway Analysis package (IPA) (www.ingenuity.com). Hierarchical clustering was conducted with Genesis (http://genome.tugraz.at/). In order to determine nonrandom distribution of significantly regulated genes, chi‐square and Pearson's Correlation Coefficient were calculated. The array data have been uploaded to GEO (accession number GSE74145).

### Intraperitoneal glucose tolerance test phenotyping

In order to evaluate glucose uptake as a first approximation of a diabetes‐like phenotype, we performed intraperitoneal glucose tolerance test (IPGTT) on male rats (*N* = 18) at 50–60 days of age that had been exposed to normoxia (control) or neonatal 10% IH. As previously described, rats were fasted overnight for 16 h prior to IPGTT in order to achieve a non‐absorptive state (Waner and Nyska 1994). Immediately after the basal blood sample was collected, glucose (1 g/kg) was administered via intraperitoneal injection in a 50% dextrose solution, diluted in sterile PBS to a final volume of 700 *μ*L per injection. Blood was collected via tail clip at 0, 15, 30, 60, 90, and 120 min post glucose injection as described previously (Chintamaneni et al. 2014). Blood samples were centrifuged and plasma frozen for subsequent corticosterone, glucose, and insulin analysis. IPGTT data were analyzed by 2‐way ANOVA repeat on one factor (time) followed by Holm‐Sidak post hoc tests. Area under the curve (AUC) above baseline was calculated using the trapezoidal rule (Sigma Stat 12.5).

### Corticosterone, glucose, and insulin assays

All analytical techniques have been published previously (Chintamaneni et al. 2013). Plasma corticosterone was measured by radioimmunoassay (MP Biomedicals, Solon, OH). Plasma glucose was quantified spectrophotometrically using the glucose oxidase method (Pointe Scientific, Canton, MI). Insulin was measured by enzyme‐linked immunosorbent assay (Crystal Chem, Downers Grove, IL).

## Results

**Figure 1:**
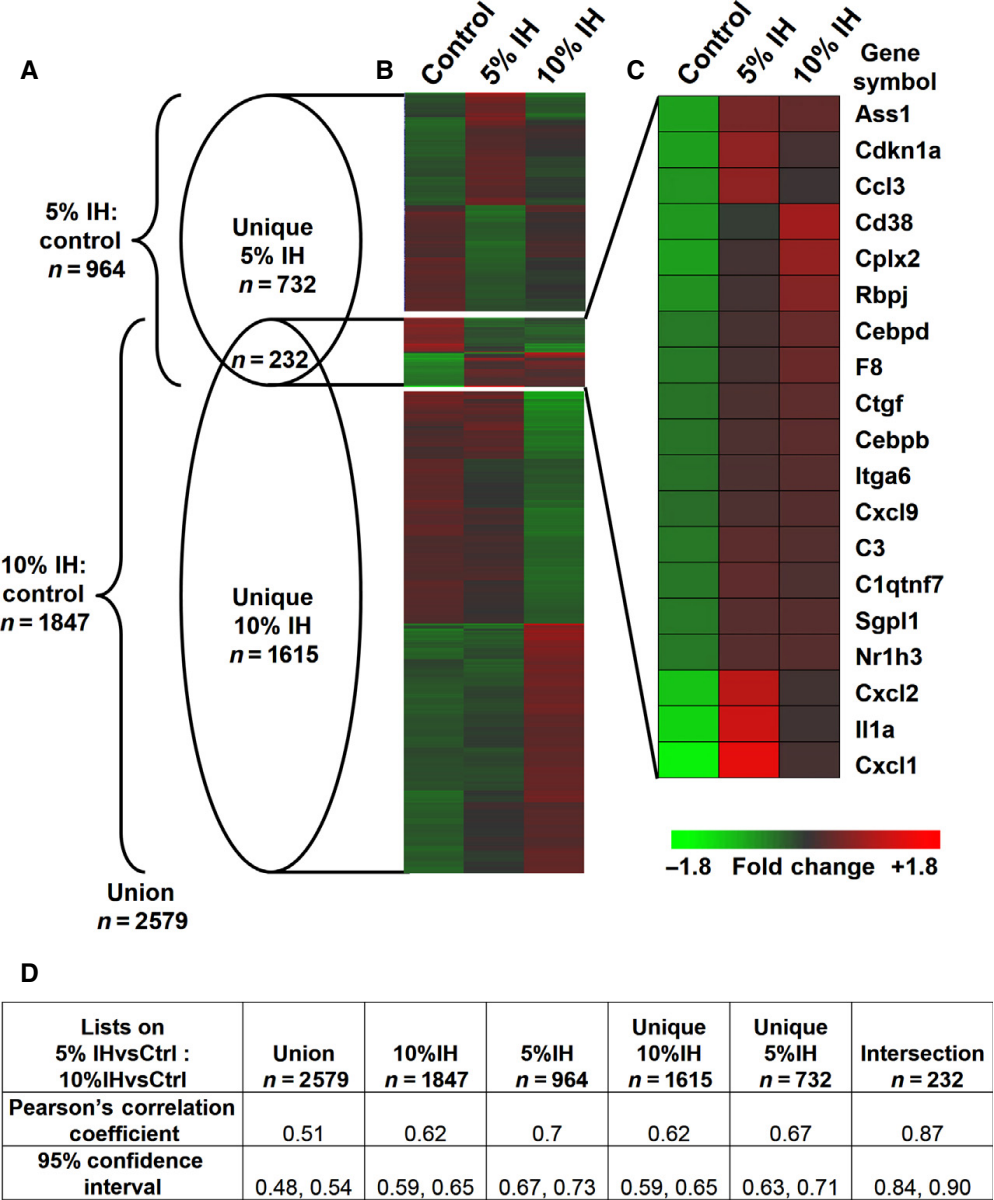
Analysis of genes significantly regulated by plasma from adult rats exposed to neonatal 5% IH and adult plasma exposed to neonatal 10% intermittent hypoxia compared to normoxic control using Genesis. (A) A Venn diagram illustrates the 2579 genes that were identified as significant using the cutoff criteria of RP *P*‐value <0.05 and ¦log_2_ ratio¦ > 0.263. (B) A heat map shows the mean expression of each experimental condition (Normoxic control vs. 5% IH vs. 10% IH) and are subdivided relative to the Venn diagram (top section = genes unique to 5% IH,* n* = 732; middle section = intersection of 5% IH vs. 10% IH,* n* = 232; bottom section = genes unique to 10% IH,* n* = 1615). (C) A subset of well‐annotated genes from the intersection of 5% IH and 10% IH plasma related to immune function are represented with a heat map. (D) Pearson's Correlation Coefficient and 95% confidence interval values are listed for all subsets of the Venn diagram.

We hypothesized that plasma‐borne factors related to IH could be detected through their ability to induce gene expression in a healthy PBMC population. Therefore, co‐cultures were prepared with plasma from adult rats exposed to neonatal IH or normoxia. The transcriptional responses of the reporter cell population were comprehensively evaluated with a high‐density microarray. Figure 1 shows the data structure of the significantly regulated genes identified when comparing plasma from adult rats exposed to neonatal 5% IH versus normoxic control and plasma from adult rats exposed to neonatal 10% IH versus normoxic control. With a significance criterion of Rank Product (RP) *P*‐value <0.05 and ¦log_2_ ratio¦ > 0.263 (1.2‐fold), a total of 2579 regulated genes were identified. In Figure 1A, a Venn diagram represents the 2579 genes that were subcategorized into (1) significantly regulated genes unique to adult plasma exposed to neonatal 5% IH treatment (*N* = 732); (2) all genes significantly regulated in the presence of adult plasma exposed to neonatal 5% IH (5% IH + intersection, *N* = 964); (3) significantly regulated genes unique to adult plasma exposed to neonatal 10% IH treatment (*N* = 1615); and (4) all genes significantly regulated in the presence of adult plasma exposed to neonatal 10% IH (10% IH + intersection, *N* = 1847). The heat map in Figure 1B illustrates the significantly annotated genes from the Venn diagram comparing normoxic control versus 5% IH and 10% IH. Figure 1C shows a subset of the well‐annotated genes from the intersection of 5% IH and 10% IH (*n* = 232) that are related to immune function. The intersection of 232 transcripts was significantly nonrandom (*χ* ^2^ = 608.2, *P* < 0.001) and found to be highly correlative (Pearson's Correlation Coefficient = 0.88). All subsets of genes were found to be positively correlative (Fig. 1D). Overall, the two data sets exhibited identity, with the 2579 probe set union showing a Pearson's Correlation Coefficient of 0.51 and the 964 transcript 5% IH data set and 1847 transcript 10% IH data set showing Pearson's Correlation Coefficients of 0.70 and 0.62, respectively.

All genes with a fold‐change of ±1.5 (or greater) from adult plasma exposed to neonatal 5% IH and adult plasma exposed to neonatal 10% IH are listed in Table 1 with their respective fold‐change values relative to autologous plasma. Many of the genes altered by adult plasma exposed to neonatal 5% IH treatment group that had the greatest fold‐changes were genes important in inflammatory and immune responses. These included *Cxcl1* (chemokine C‐X‐C motif ligand 1)*, Cxcl2* (Chemokine C‐X‐C motif ligand 2)*, Ccl3* (Chemokine C‐C motif ligand 3) *Il1a* (Interleukin 1, alpha), and *Il1b* (Interleukin 1, beta), all of which had a fold change greater than 1.5.

**Table 1:** Significantly regulated genes induced by plasma from adult rats previously exposed to neonatal 5% or 10% IH

Gene symbol	Gene name	Fold‐change
*Genes significantly regulated with 5% IH*		
*Cxcl1*	Chemokine (C‐X‐C motif) ligand 1	2.50
*Il1a*	Interleukin 1, alpha	2.34
*Ptgs2*	Prostaglandin‐endoperoxide synthase 2	2.25
*Il1b*	Interleukin 1, beta	1.97
*Cxcl2*	Chemokine (C‐X‐C motif) ligand 2	1.92
*Lcn2*	Lipocalin 2	1.64
*Siglec5*	Sialic acid binding lg‐like lectin 5	1.61
*Ptges*	Prostaglandin E synthase	1.59
*Cdkn1a*	Cyclin‐dependent kinase inhibitor 1A	1.58
*Ccl3*	Chemokine (C‐C motif) ligand 3	1.53
*Pdgfa*	Platelet‐derived growth factor alpha polypeptide	1.50
*Abca5*	ATP‐binding cassette, sub‐family A, member 5	−1.53
*Genes significantly regulated with 10% IH*		
*Rasd1*	RAS, dexamethasone‐induced 1	2.91
*Dhtkd1*	Dehydrogenase E1+ transketolase domain containing 1	1.82
*Sfrs2ip*	SR‐related CTD‐associated factor 11	1.78
*Cxcl1*	Chemokine (C‐X‐C motif) ligand 1	1.68
*Gadd45a*	Growth arrest & DNA damage inducible alpha	1.65
*Cd38*	CD38 molecule	1.62
*Cish*	Cytokine inducible SH2‐containing protein	1.61
*Cplx2*	Complexin 2	1.59
*Dck*	Deoxycytidine kinase	1.57
*Fkbp5*	FK506 binding protein 5	1.56
*Cul5*	Cullin 5	1.56
*Zfp317*	Zinc finger protein 317	1.54
*Rbbp6*	Retinoblastoma binding protein 6	1.54
*Pde4d*	Phosphodiesterase 40, cAMP specific	1.53
*Rif1*	Replication timing regulatory factor 1	1.53
*Api5*	Apoptosis inhibitor 5	1.53
*Pdha1*	Pyruvate dehydrogenase (lipamide) alpha 1	1.52
*Atxn3*	Ataxin 3	1.51
*Il1a*	Interleukin 1, alpha	1.51
*Fcer2*	Fc fragment of IgE, low affinity II, receptor for CD23	−1.53
*Serpine2*	Serpin peptidase inhibitor, clade E, member 2	−1.53
*Cbl*	Cbl proto‐oncogene E3, ubiquitin protein ligase	−1.53
*Igha*	Immunoglobin heavy constant alpha	−1.54
*Jam2*	Junction adhesion molecule 2	−1.55
*Klhl14*	Kelch‐like family member 14	−1.56
*Usp7*	Ubiquitin specific peptidase 2	−1.61
*Eif2c2*	Eukayotic translation initiation factor 2C, 2	−1.64
*Zfp451*	Zinc finger protein 451	−1.70
*Plscr1*	Phospholipid scramblase	−1.72
*Ubd*	Ubiquitin D	−1.73

Genes shown have a fold change ±1.5 or greater compared to autologous plasma (background).

Interestingly, these inflammatory genes (*Cxcl1, Cxcl2, Ccl3, Il1b*) did not have the same response in the adult plasma exposed to neonatal 10% IH. *Il1a* was the only inflammatory gene significantly upregulated with adult plasma exposed to neonatal 5% IH that was also significantly upregulated with neonatal 10% IH.

Tables 2 and 3 list the DAVID pathway analyses for data from the adult plasma exposed to neonatal 5% IH and 10% IH treatment groups. Each group was subcategorized into four groups for analysis: all genes from each treatment (adult plasma exposed to neonatal 5% IH or 10% IH), genes unique to each treatment, only upregulated genes, and only downregulated genes. The most affected biological pathways, according to *P*‐value, with the 5% neonatal IH treatment are shown in Table 2. The pathway analysis revealed inflammatory response, cytokine activity, and immune response as some of the most significantly upregulated biological pathways, with genes specifically related to the IL‐1 pathway. All of the most downregulated pathways were related to homeostatic processes.

**Table 2:** Significantly regulated biological processes induced by plasma from adults previously exposed to neonatal 5% IH

Biological process	Count	Percentage	*P* value
*Significant pathways regulated with 5% IH*			
Response to wounding	40	5.9	1.00E‐08
Plasma membrane	137	20.2	1.80E‐08
Extracellular space	44	6.5	1.10E‐07
Inflammatory response	25	3.7	2.80E‐07
Defense response	35	5.2	5.80E‐07
Cytokine activity	17	2.5	1.00E‐06
Immune response	35	5.2	1.30E‐06
*Significant pathways unique to 5% IH*			
Plasma membrane	111	21.4	2.60E‐08
Inflammatory response to antigenic stimulus	6	1.2	2.70E‐06
Immune response	29	5.6	4.30E‐06
Inflammatory response	20	3.9	4.30E‐06
*Significant pathways upregulated with 5% IH*			
Defense response	31	8.9	2.20E‐11
Inflammatory response	22	6.3	2.90E‐10
Response to wounding	29	8.3	2.10E‐09
Immune response	28	8	5.50E‐09
Cytokine activity	15	4.3	1.20E‐08
Response to bacterium	19	5.4	4.40E‐08
*Significant pathways downregulated with 5% IH*			
Cation homeostasis	18	5.4	9.10E‐07
Cellular, di‐, tri‐valent inorganic cation homeostasis	15	4.5	5.40E‐06
Chemical homeostasis	23	6.9	1.10E‐05
Cellular cation homeostasis	15	4.5	2.10E‐05
Homeostatic process	27	8.1	6.20E‐05

Genes identified in Figure 1 were analyzed by DAVID to determine significantly regulated biological processes, and the most significant pathways regulated by 5% IH, determined by *P*‐value, are listed. The count value indicates the number of genes identified in that particular pathway, the percentage indicates the total number of genes in that pathway from the entire list submitted, and the *P*‐value defines the significance of the association of that particular pathways with the given gene list that was analyzed.

**Table 3:** Significantly regulated biological processes induced by plasma from adult rats previously exposed to neonatal 10% IH

Biological process	Count	Percentage	*P* value
*Significant pathways regulated with 10% IH*			
Platelet alpha granule	10	2	4.20E‐06
Basement membrane	11	2.3	8.10E‐06
Response to abiotic stimulus	26	5.3	1.10E‐05
Secretory granule	16	3.3	1.40E‐04
MAPK signaling pathway	17	3.5	5.60E‐04
Regulation of body fluid levels	10	2	7.00E‐04
Coagulation	8	1.6	9.90E‐04
*Significant pathways unique to 10% IH*			
Axon	11	4.3	2.00E‐04
Intrinsic to plasma membrane	14	5.5	1.10E‐03
MAPK signaling pathway	11	4.3	2.70E‐03
B cell receptor signaling pathway	6	2.4	3.20E‐03
Gland morphogenesis	6	2.4	4.30E‐03
*Significant pathways upregulated with 10% IH*			
Platelet alpha granule	9	3.2	9.50E‐07
Cytoplasmic vesicle part	13	4.6	8.20E‐06
Basement membrane	9	3.2	1.00E‐05
Secretory granule	14	4.9	1.10E‐05
Response to wounding	18	6.3	7.30E‐05
Regulation of body fluid levels	9	3.2	7.30E‐05
Coagulation	7	2.5	2.80E‐04
*Significant pathways downregulated with 10% IH*			
Phosphoprotein	52	25.1	2.50E‐07
Chromatin binding	10	4.8	2.70E‐05
MAPK signaling pathway	12	5.8	2.90E‐05
Regulation of RNA metabolic process	29	14	5.40E‐05
Natural killer cell mediated cytotoxicity	7	3.4	3.30E‐04
DNA binding	28	13.5	6.20E‐04
B cell receptor signaling pathway	6	2.9	6.60E‐04

Genes identified in Figure 1 were analyzed by DAVID to determine significantly regulated biological processes, and the most significant pathways regulated by 10% IH, determined by *P*‐value, are listed. The count value indicates the number of genes identified in that particular pathway, the percentage indicates the total number of genes in that pathway from the entire list submitted, and the *P*‐value defines the significance of the association of that particular pathways with the given gene list that was analyzed.

Table 3 lists the significantly regulated biological pathways from the adult plasma exposed to neonatal 10% IH treatment group. While inflammatory‐related pathways were the most significant pathways regulated with 5% IH, the inflammatory response only had a *P*‐value of 5.4E‐02 containing only 0.9% of the genes from the 10% IH gene list (data not shown).

To identify candidate mediators underlying the observed signatures, we utilized the Ingenuity Pathway Analysis (IPA) upstream regulator tool. Significantly regulated pathways with adult plasma exposed to neonatal 5% IH included granulocyte and agranulocyte adhesion and diapedesis, IL‐10 signaling, and TREM1 signaling. Significant upstream regulators included IL1B and MYD88, an essential signal transducer in the IL‐1 pathway. Significant pathways with 10% IH included p53 signaling, death receptor signaling, and agranulocyte adhesion and diapedesis. The top upstream regulators included TGFB1, IL3, and IL7.

**Figure 2:**
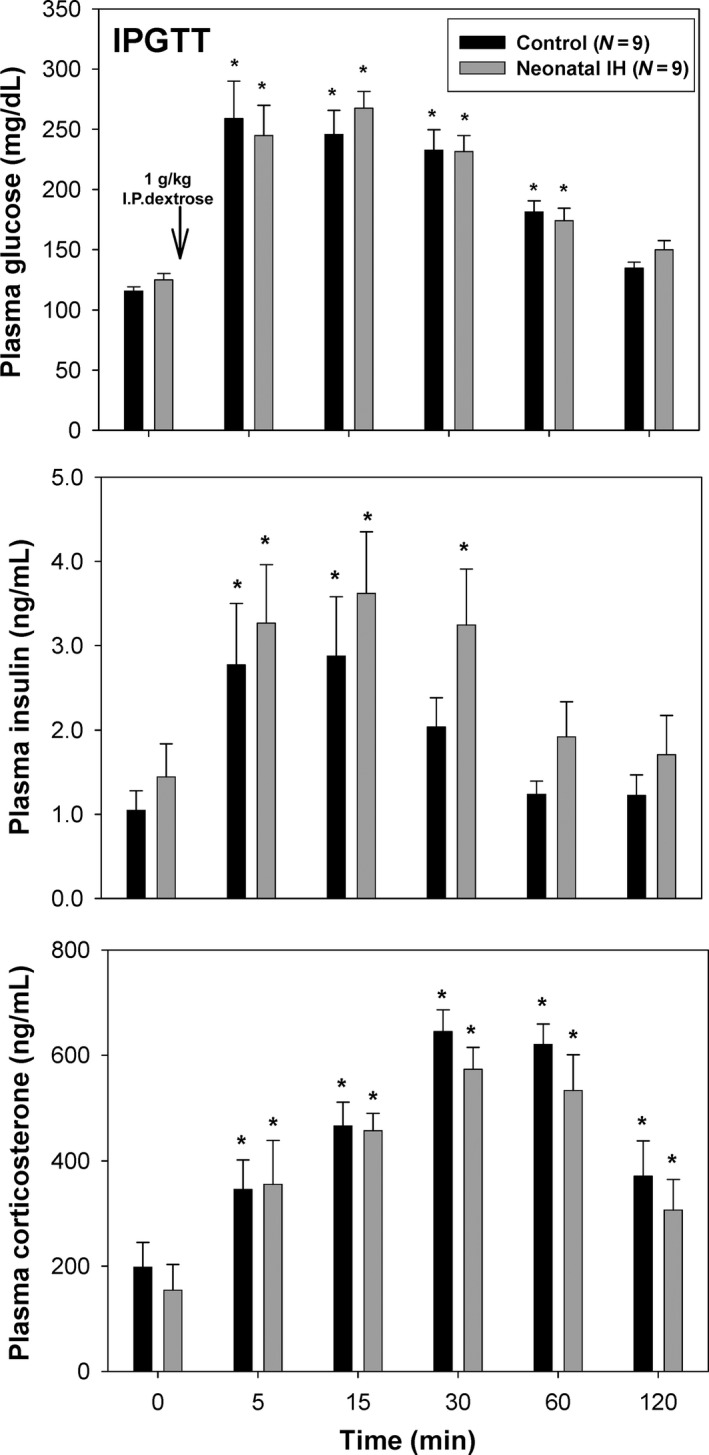
Intraperitoneal glucose tolerance test (IPGTT) in adult rats exposed to neonatal 10% IH versus normoxic control. Plasma glucose, insulin, and corticosterone were measured at preglucose injection (time = 0), and at 5, 15, 30, 60, and 120 min postglucose injection. *N* = 9 rats per mean ± SEM. *, Significant difference from baseline.

The set of plasma‐induced transcription experiments defined signatures showing significant regulation of genes responsible in inflammatory response pathways, specifically the IL‐1 pathway, suggesting neonatal exposure to intermittent hypoxia may play a role in the development of late‐onset inflammatory diseases (Tables 1, 2, 3). Additionally, these results comparing plasma from adult rats exposed to neonatal 5% IH to 10% IH suggested a transcriptional signature that included a significant innate immune component similar to diabetic rats (Kaldunski et al. 2010). It is known that increased cytokine levels can impair beta cell function, therefore we hypothesized that neonatal IH exposure may result in glucose intolerance as adults. Figure 2 shows the intraperitoneal glucose tolerance test (IPGTT) results conducted on adult rats previously exposed to 10% IH as neonates. Plasma glucose concentrations were not significantly different between adults exposed to neonatal IH and normoxic control rats. Plasma corticosterone from adult rats exposed to IH as neonates tended to be lower at each time point compared to the control; however, this was not significant. Plasma insulin levels tended to be higher in the adults exposed to neonatal IH, however, this was not significantly different from the control. The AUCs of glucose were 22717.4 ± 891.3 mg/dL/120 min (control) and 23030.2 ± 892.5 mg/dL/120 min (10% IH) (*N* = 9, *P* = 0.807). The AUCs of insulin were 197.5 ± 34.5 ng/mL/120 min (control) and 283.9 ± 60.1 ng/mL/120 min (10% IH). There was a tendency for the AUC of insulin to be greater with 10% IH; however, this was not significant (*N* = 9, *P* = 0.230).

## Discussion

We hypothesized that exposure to *neonatal* intermittent hypoxia would exhibit an upregulation of inflammatory genes and pathways in the *adult* rat from long‐lasting programming. Using plasma‐induced transcriptional analysis, we determined that the extracellular milieu of adult rats previously exposed to neonatal intermittent hypoxia (5% or 10% O_2_) possesses a proinflammatory bias. Of most interest was the transcriptional increase of *Il1a* and *Il1b* induced by plasma from adults exposed to neonatal 5% IH and 10% IH. IL‐1 plays a central role in the regulation of immune and inflammatory responses. Furthermore, increased basal IL‐1*α* and IL‐1*β* expression levels can lead to the development of the type 2 diabetes mellitus phenotype (Banerjee and Saxena 2012).

The neonatal rat is a useful model for human prematurity because it is an altricial species; the PD12 rat is considered similar neurologically and physiologically to a full term human neonate (Romijn et al. 1991; Clancy et al. 2001, 2007; Guenther et al. 2012; Chintamaneni et al. 2013, 2014). Therefore, discussion of the long‐term effects of late gestation fetal hypoxia is worthwhile in this context, particularly because studies of the long‐term effects of fetal hypoxia are more numerous and comprehensive than neonatal hypoxia in precocial species.

There is a general consensus that prenatal hypoxia exposure leads to an increased risk of insulin resistance and altered glucose metabolism in adulthood (Camm et al. 2011; Rueda‐Clausen et al. 2011; Cao et al. 2012), and an increased risk to develop diabetes (Coughlan et al. 2004; Clarenbach et al. 2011). Cao et al. showed that prenatal hypoxia damages the developing hepatic parenchyma cells that are associated with hepatic insulin signaling (Cao et al. 2012). This leads to altered glucose metabolism, insulin sensitivity, and an increase in inflammatory‐related diseases in postnatal life. Camm et al. proposed that the mechanism of insulin resistance in adults exposed to fetal hypoxia is through the PI3K/Akt signaling cascade, specifically through the decrease in expression of Akt‐2 in muscle and liver tissue (Camm et al. 2011). Akt‐2 plays a key role in the signal transduction downstream of the insulin receptor, and they showed that Akt‐2 was significantly decreased in the muscle tissue of adults exposed to fetal hypoxia compared to normoxic controls. Additionally, Akt‐2 knockout mice have a profound diabetic phenotype and severe insulin resistance (Cho et al. 2001). However, we found no significant difference in Akt‐2 expression in either treatment group (adult plasma from rats exposed to neonatal 5% IH or adult plasma from rats exposed to neonatal 10% IH).

We have previously shown that neonatal IH exposure has a programming effect on the HPA axis, resulting in an augmentation of plasma ACTH levels, and a prolonged corticosterone response to restraint stress (Chintamaneni et al. 2013). Nesterenko et al. described links between fetal programming and adult onset inflammatory diseases, but the mechanism is unclear (Nesterenko and Aly 2009). However, they conclude that adult disease onset is not an effect of just fetal hypoxia exposure alone, but rather a combined effect of oxidative stress, nutrient uptake, birth weight, immune maturation, and gene expression. One study showed that chronic fetal hypoxia exposure resulted in an upregulation of inflammatory related genes, while acute fetal hypoxia exposure only showed an upregulation of hexokinase, phosphofructokinase, and aldolase related genes (Huang et al. 2004).

There were interesting differences in the signature between adult rats exposed to 5% and 10% neonatal IH. Of most interest was the difference in inflammatory markers between 5% IH and 10% IH. There was a significant increase in inflammatory‐related genes (*Cxcl1, Cxcl2, Ccl3, Il1b)* and inflammatory‐related pathways with adult rats exposed to neonatal 5% intermittent hypoxia. These genes did not show a significant increase in expression with 10% intermittent hypoxia. We have observed a similar phenomenon with intermittent neonatal hypoxia with respect to the behavior of the HPA axis in adults (Chintamaneni et al. 2014). In that study, less severe intermittent hypoxia (10% O2) produced a different HPA axis “programming” response than more severe intermittent hypoxia (5% O2). Furthermore, the neonatal rat has a hypothermic response (Guenther et al. 2012) which is likely to be a covariate in the acute and long‐term effects of neonatal hypoxia. We are currently evaluating the long‐term effects in adult rats after exposure to neonatal hypo‐ versus isothermia during neonatal hypoxia.

To put these hypoxic exposures into context, we have shown previously that 10% IH produces trough transcutaneous O2 levels of about 40–50% and 5% IH produces trough transcutaneous O2 levels of about 25–30% (Chintamaneni et al. 2014). This is relevant clinically as human premature neonates can achieve these severe levels of hypoxia during periods of apnea (Hiatt et al. 1981; Al‐Matary et al. 2004).

There are few studies on the programming effects of neonatal intermittent hypoxia on adult metabolism. Since neonatal IH increases plasma corticosterone concentrations (Chintamaneni et al. 2013), it is useful to briefly discuss the long‐term effects of neonatal glucocorticoid (GC) treatment. It is known that GCs play a major role in immune development (King et al. 1995). It is assumed that GC treatment during the first few years of life has an effect on immune development since it is noted that children receiving the treatment have increased hospital admissions for infections compared to nontreated children of the same age (Smolders‐de et al. 1990). Part of this may be due to the fact that preterm infants are commonly treated with high doses of GC (Cummings et al. 1989). Early neonatal GC exposure has permanent programming effects on immunocompetence and neuroendocrine functioning later in life (Bakker et al. 2000). In rats neonatally treated with dexamethasone, adults had a decreased production of cytokines and exhibited a shift toward pro‐inflammatory cytokine profile (Bakker et al. 2000). This is the same as observed in 7–10 year old children that had neonatal GC treatment (Karemaker et al. 2008).

Although the transcriptional signature highlights the upregulation of the IL‐1 pathway and subsequent inflammatory markers, we did not show an effect of neonatal IH on the plasma glucose, insulin, and corticosterone responses to IP glucose. It is possible that the glucose tolerance test is too potent a stimulus to detect subtle changes in insulin responsiveness and sensitivity. For logistical reasons, we performed the IPGTT in the rats when they were younger compared to when the plasma was obtained for signature analysis. It is possible that older rats may have shown significant glucose insensitivity. However, Pae et al. performed glucose tolerance tests just a few days after neonatal IH exposure (Pae et al. 2013). They reported abnormal blood glucose regulation in IH treated animals compared to control, where blood glucose initially increased at a much faster rate and peaked significantly higher than the control group. Plasma insulin levels were also significantly lower in the IH treated group. Despite these caveats, the transcriptional signature we identified may not be related to a long‐term change in glucose regulation.

## Conclusions

We have identified a novel plasma‐induced transcriptional signature in adult rats exposed to neonatal 5% and 10% intermittent hypoxia that contains an upregulation of inflammatory‐related genes, most interestingly, *Il1a* and *Il1b*. Although this transcriptional signature did not translate to a phenotypic change in glucose regulation in adults exposed to neonatal intermittent hypoxia compared to normoxic control, future experiments will evaluate glucose regulation using a more subtle stimulus (Sjostrand et al. 2014; Ye et al. 2014, 2015). Lastly, the phenotypic focus for this study was on glucose regulation as well as the circulating milieu in adult rats that experienced IH as neonates. There is the potential that this transcriptional signature may uncover changes in other pathophysiological systems important in late‐onset inflammatory diseases.

## Conflict of Interest

The authors have no conflicts of interest to disclose.
